# A dose-response relationship between exposure to a large-scale HIV preventive intervention and consistent condom use with different sexual partners of female sex workers in southern India

**DOI:** 10.1186/1471-2458-11-S6-S8

**Published:** 2011-12-29

**Authors:** Kathleen N Deering, Marie-Claude Boily, Catherine M Lowndes, Jean Shoveller, Mark W Tyndall, Peter Vickerman, Jan Bradley, Kaveri Gurav, Michael Pickles, Stephen Moses, Banadakoppa M Ramesh, Reynold Washington, S Rajaram, Michel Alary

**Affiliations:** 1 Division of AIDS, Department of Medicine, Faculty of Medicine, University of British Columbia, Vancouver, Canada; 2 Department of Infectious Diseases Epidemiology, Imperial College, London, UK; 3 HIV and STI Department, Health Protection Services – Colindale, Health Protection Agency, London, UK; 4 Faculty of Medicine, University of Ottawa, Ottawa, Canada; 5 London School of Hygiene and Tropical Medicine, London, UK; 6 URESP, Centre de recherche FRSQ du CHA universitaire de Québec, Québec, Canada; 7 Karnataka Health Promotion Trust, Bangalore, India; 8 Department of Medical Microbiology, University of Manitoba, Winnipeg, Canada; 9 Department of Community Health Sciences, University of Manitoba, Winnipeg, Canada; 10 Département de médecine sociale et préventive, Université Laval, Québec, Canada; 11 Institut national de santé publique du Québec, Québec, Canada

## Abstract

**Background:**

The *Avahan Initiative*, a large-scale HIV preventive intervention targeted to high-risk populations including female sex workers (FSWs), was initiated in 2003 in six high-prevalence states in India, including Karnataka. This study assessed if intervention exposure was associated with condom use with FSWs’ sexual partners, including a dose-response relationship.

**Methods:**

Data were from a cross-sectional study (2006-07) of 775 FSWs in three districts in Karnataka. Survey methods accounted for the complex cluster sampling design. Bivariate and multivariable logistic regression was used to separately model the relationships between each of five intervention exposure variables and five outcomes for consistent condom use (CCU= always versus frequently/sometimes/never) with different sex partners, including with: all clients; occasional clients; most recent repeat client; most recent non-paying partner; and the husband or cohabiting partner. Linear tests for trends were conducted for three continuous intervention exposure variables.

**Results:**

FSWs reported highest CCU with all clients (81.7%); CCU was lowest with FSWs’ husband or cohabiting partner (9.6%). In multivariable analysis, the odds of CCU with all clients and with occasional clients were 6.3-fold [95% confidence intervals, CIs: 2.8-14.5] and 2.3-fold [95% CIs: 1.4-4.1] higher among FSWs contacted by intervention staff and 4.9-fold [95% CIs: 2.6-9.3] and 2.3-fold [95% CIs: 1.3-4.1] higher among those who ever observed a condom demonstration by staff, respectively, compared to those who had not. A significant dose-response relationship existed between each of these CCU outcomes and increased duration since first contacted by staff (*P*=0.001; *P*=0.006) and numbers of condom demonstrations witnessed (*P*=0.004; *P*=0.026); a dose-response relationship was also observed between condom use with all clients and number of times contacted by staff (*P*=0.047). Intervention exposure was not associated with higher odds of CCU with the most recent repeat client, most recent non-paying partner or with the husband or cohabiting partner.

**Conclusion:**

Study findings suggest that exposure to a large-scale HIV intervention for FSWs was associated with increased CCU with commercial clients. Moreover, there were dose-response relationships between CCU with clients and increased duration since first contacted by staff, times contacted by staff and number of condom demonstrations. Additional program effort is required to increase condom use with non-commercial partners.

## Background

Sex work-related harms are linked inextricably to the social, economic, policy, and physical environments of sex workers. Individual behaviour (high- or low-risk) both shapes and is shaped by individual and environmental factors [1,2]. There has thus been increasing recognition of the importance of using structural and community-level strategies that modify sex work environments to reduce risk and promote health among sex workers and their clients, and in particular, improve condom use with sex partners [3-6]. Notably, in response to high rates of HIV and sexually transmitted infections (STIs) among female sex workers (FSWs) in the early 1990s, several countries in east Asia instituted a 100% condom use campaign intended to increase social acceptance of condoms, influence men to agree to use condoms and empower FSWs to demand condom use with clients, as well as increase access to STI testing and treatment. This programme is thought to have contributed to dramatic declines in STIs and HIV in Thailand and Cambodia, as well as influence similar campaigns across Asia [7-9]. The Sonagachi Project in Kolkata, India, implemented a community empowerment model for FSWs that framed health risks to sex workers as occupational hazards, focusing on addressing community- and individual-level factors influencing risk for HIV. Subsequently, large increases in condom use have been observed and HIV prevalence remains low in FSWs associated with the Sonagachi Project [10,11].

Another large-scale intervention designed to reduce HIV infection rates among groups with high HIV risk (FSWs; men-who-have-sex-with-men; injection drug users) and groups that bridge high- and low-risk groups (clients of FSWs) is Avahan, the India AIDS Initiative [12,13], which began in 2003 in the six states with the highest HIV prevalence in India. Using community involvement and mobilization strategies, combined with condom promotion and increased STI clinical services among these populations, the ongoing Avahan AIDS Initiative addresses proximal and distal determinants of risk. The Avahan AIDS Initiative aims to increase condom use among groups at high risk for HIV by modifying their environments to enable individuals to use condoms [14]. For FSWs, this is achieved through a combination of approaches. Avahan includes peer-led outreach to increase awareness of condoms and ability to negotiate condom use with clients [15] and efforts to increase the availability of and access to condoms and STI testing and treatment centres [12]. The program also includes actions to improve community mobilization and involvement. FSWs have played important roles in mapping local hotspots, informing outreach plans, developing peer networks in communities and participating in training and implementation of Avahan surveys [16,17]. The program has also supported the development and operation of safer sex work spaces, including sex work drop-in centres and collectives, where women can rest safely, take classes (e.g., literacy training) and interact with staff and other FSWs [18-20]. Legal empowerment training has also been offered to 25,000 FSWs across Karnataka state, to improve legal literacy and inform FSWs about their legal rights [19]. The evaluation of this large-scale intervention remains challenging, as is the case for many similar evaluation efforts where conventional methods (e.g., randomized control trials of communities) are unethical and/or impractical to implement [13,21]. A multi-pronged evaluation framework is necessary to gain an overall understanding of an intervention’s impact [21]. This includes an examination of programmatic (e.g., numbers of peer educators, clinics or services to meet the population’s needs) and health indicators (e.g., increases in condom use, decreases in HIV or STI incidence). The consistency of study results from a combination of study designs, including transmission dynamics modelling (e.g., testing hypotheses while taking into account uncertainty in parameter estimates), cost-effectiveness analysis, surveillance and epidemiological approaches, can together provide a stronger understanding of the effectiveness of the intervention [22].

As part of this comprehensive evaluation framework, the objective of the current analysis was to determine if the Avahan AIDS Initiative had an impact on condom use amongst FSWs in urban areas of three districts in Karnataka State, India. HIV prevalence among FSWs was 12.7% in Bangalore district, 15.7% in Bellary and 33.9% in Belgaum in the mid-2000s [23]. Specifically, we assessed whether five variables measuring intervention exposure were associated with consistent condom use (CCU) (i.e. 100%) among FSWs with: (1) all clients on the most recent day worked; (2) their current occasional clients (i.e., clients who FSWs are not familiar with and who visit FSWs once); (3) their most recent repeat client (i.e., regular clients who FSWs are familiar with and who visit FSWs more than once); (4) their most recent non-paying partner and (5) their husband or cohabiting partner.

## Methods

### Study design and sampling

During 2006-07, in-depth face-to-face interviews (Special Behavioural Surveys, SBS) were conducted with 775 FSWs in three districts in Karnataka state, located in southern India. A probability sampling method was employed, using time-location cluster sampling with normalized weights calculated to account for the complex sampling design. Sampling methods were similar to those reported by Ramesh et al [23] for other studies carried out among FSWs in Karnataka state.

### Survey organization and methods

The surveys were implemented by the CHARME-India project in collaboration with the Institute of Population Health and Clinical Research (IPHCR), St John’s Medical College, and the Karnataka Health Promotion Trust (KHPT), Bangalore, India, the Centre hospitalier *affilié* universitaire de Québec (CHA), Québec, Canada, and the University of Manitoba, Winnipeg, Canada. The surveys were administered face-to-face by trained interviewers in the local language (*Kannada*) and were conducted anonymously, with no names or personal identifiers recorded. Ethics approval was attained from the CH*A* and the University of Manitoba as well as St. John’s Medical College.

### Outcomes

The first outcome, CCU with all clients (including both occasional and repeat) during all instances of sexual intercourse in the most recent day worked was derived by dividing the reported number of instances of sexual intercourse in which condoms were used by the reported total number of instances of sexual intercourse in the most recent day worked. This was used to create a dichotomized variable of 100% versus <100% of instances of sexual intercourse in which condoms were used. The remaining four outcomes described CCU with FSWs’ different sexual partners, including: commercial sex clients (their current occasional clients; their most recent repeat client); and non-commercial partners (their most recent non-paying partner who was neither a husband nor the main cohabiting partner; and their husband or main cohabiting partner (if they had one)). These outcomes were derived from survey items about general condom frequency with each type of partner (e.g. “How often do you use condoms with <this partner>?”). Condom use was considered to be CCU with their partners, if they answered ‘always’, as opposed to ‘often’, ‘sometimes’ or ‘never’.

### Explanatory factors

We examined five variables measuring exposure to the intervention, including: if FSWs had been contacted by intervention staff; if FSWs had been given condoms by intervention staff; the duration of time since contacted by intervention staff (years), which was specific to each district and limited to the total number of years women could have been exposed to the intervention (the year/month of the start of the intervention subtracted from the year/month of the survey – 1.5-2.5 years); the number of times in the past month FSWs had been contacted by intervention staff; and the number of condom demonstrations by intervention staff that FSWs had seen in the past month.

For each model, we adjusted for social and environmental factors that may influence condom use. Social factors included age, marital status (married versus unmarried, including those of the *Devadasi* tradition, a form of temple-based sex work whereby women are dedicated through marriage to gods or goddesses) [24-26], age at first sex, age at first sex work and duration of sex work. Environmental factors included district of residence, education (literacy) and having sex work as sole income (no other paid work versus any, including non-agricultural labour, petty business, maid servant, agricultural labour, handicrafts and other). It also included FSWs’ working environment, which was represented by the type of solicitation (independent or through a middleman/pimp) and the place of solicitation of clients of FSWs, which was grouped into three categories: home-based (home; rented room), brothel-based (lodge; *dabha* [road-side lodge-type establishment]; brothel); and public-places-based (vehicle; bar/night club; public places, such as bus stops, train stations and the street).

### Statistical analysis

Statistical analysis was conducted using survey methods in SAS Version 9.1 [27], taking into account the sampling clusters and weights. FSWs sampled from the same clusters are assumed to be more similar to each other than they are to FSWs from different clusters; survey methods account for this by estimating the overall variance from the variation among the clusters [28]. Descriptive statistics were calculated for sample characteristics. The prevalence of outcomes was calculated for each variable describing exposure to the intervention. Bivariate and multivariable logistic regression was used to model the relationship between the condom use outcomes and the five variables describing exposure to the intervention. Five separate logistic models were created for each of the five dichotomous outcomes, for a total of 25 separate models. Inclusion into multivariable models was based on significance at the P<0.10-level from Wald chi-squared tests in bivariate regression analyses, or if they were perceived to be important confounders *a priori* (district, typology of sex work). Each single intervention variable was forced into the five different multivariable models to examine the independent relationship between intervention exposure and CCU. Two intervention exposure variables were dichotomous (ever been contacted by intervention staff, ever seen a condom demonstration by intervention staff), while three were originally continuous (duration since first contacted by intervention staff, number of times contacted by intervention staff, number of condom demonstrations given by staff). The continuous variables were categorized prior to analysis. To examine a dose-response relationship, a linear test for trends across categories for each of the three continuous intervention exposure variables and each CCU outcome was conducted. The median of each category was taken, and the exposure variable was treated as a continuous variable. Odds ratios (ORs) and adjusted odds ratios (AORs) and their 95% confidence interval [95% CIs] were reported for logistic regression and *P*-values were reported for the tests for trends. All *P*-values reported are two-sided.

## Results

### Sample characteristics

Table 1 presents characteristics of the overall sample of FSWs in three districts in Karnataka state. The sample sizes for Belgaum, Bellary and Bangalore districts were 208, 198 and 369 (N=775) respectively, and the median age of FSWs across the three districts was 30 years (interquartile range [IQR]=25-35; mean=30.3 years). Of the total sample, the majority of women, 348 (55.6%), primarily solicited clients in public places, while 245 (26.2%) solicited clients from their homes and 182 (18.2%) women solicited clients in brothels. Overall, 371 (52.5%) women in the sample were divorced, separated or widowed, 229 (26.0%) were currently married, 119 (15.5%) were *Devadasi* and 56 (5.9%) were other women who were never married (Table 1).

**Table 1:** Sample characteristics and bivariate associations (unadjusted odds ratios [OR]) and 95% confidence intervals (CIs): sample characteristics and bivariate associations between social, environmental and intervention exposure factors and consistent condom use with commercial sex clients^1,2,3^

	Proportion (n) or mean /median (interquartile range=IQR) N=775	OUTCOMES					
		
		Condoms used in each occasion of sexual intercourse with all clients in the most recent day worked		Consistent condom use with occasional clients		Consistent condom use with most recent repeat client	
		
		OR [95% CIs]	P	OR [95% CIs]	P	OR [95% CIs]	P
**SOCIAL**							

Age (years)	30.3/ 30 (25-35)	1.00 [0.97-1.03]	0.935	0.97 [0.93-1.00]	0.081	0.97 [0.93-1.02]	0.188

Marital status Devadasi Never married Divorced/Separated/Widowed Currently married	15.5 (119) 5.9 (56) 52.5 (371) 26.0 (229)	1.73 [0.73-4.08] 0.65 [0.26-1.63] 0.93 [0.49-1.76] 1.0 (ref)	0.268	2.27 [1.18-4.34] 1.02 [0.47-2.22] 0.94 [0.54-1.64] 1.0 (ref)	0.031	1.30 [0.64-2.66] 1.26 [0.50-3.18] 0.77 [0.28-2.09] 1.0 (ref)	0.729

Religion Hindu (versus other - Islam/Christian/Jain)	89.1 (682)	1.01 [0.51-1.98]	0.981	1.27 [0.69-2.33]	0.445	0.53 [0.20-1.41]	0.206

Age at first sex (years)	15.5/ 15 (14-17)	1.12 [1.01-1.24]	0.036	1.07 [0.94-1.22]	0.295	1.06 [0.98-1.16]	0.137

Age at first sex work (years)	23.8/ 23 (18-29)	0.99 [0.96-1.03]	0.734	0.96 [0.93-0.99]	0.006	0.97 [0.92-1.03]	0.269

Duration of sex work (years)	6.5/ 5 (2-10)	1.01 [0.96-1.05]	0.744	1.00 [0.95-1.06]	0.892	0.99 [0.95-1.04]	0.786

**ENVIRONMENTAL**							

District Belgaum Bellary Bangalore	26.8 (208) 25.6 (198) 47.6 (369)	0.54 [0.30-1.00] 0.82 [0.41-1.63] 1.0 (ref)	0.124	1.34 [0.81-2.23] 1.58 [0.91-2.73] 1.0 (ref)	0.237	0.89 [0.32-2.43] 2.50 [0.89-7.04] 1.0 (ref)	0.005

Literate (versus cannot read/write)	27.2 (227)	1.47 [0.84-2.58]	0.177	1.67 [1.05-2.64]	0.029	2.27 [1.02-5.05]	0.044

Sex work sole income (versus has other paid work)	35.0 (301)	0.73 [0.45-1.17]	0.186	0.86 [0.57-1.30]	0.468	0.75 [0.32-1.78]	0.519

Independent solicitation (versus solicitation by a manager)	77.4 (555)	0.84 [0.47-1.48]	0.543	0.89 [0.54-1.47]	0.640	1.38 [0.52-3.65]	0.523

Typology Brothel Public places Home	18.2 (182) 55.6 (348) 26.2 (245)	0.55 [0.28-1.06] 0.93 [0.52-1.65] 1.0 (ref)	0.161	1.09 [0.58-2.07] 0.68 [0.41-1.11] 1.0 (ref)	0.142	0.88 [0.42-1.83] 0.66 [0.25-1.71] 1.0 (ref)	0.689

**INTERVENTION EXPOSURE**							

Ever contacted by intervention staff (versus not ever contacted)	85.5 (632)	2.88 [1.56-5.32]	<0.001	2.23 [1.31-3.82]	0.003	1.06 [0.42-2.68]	0.901

Had a condom demonstration by intervention staff (versus never had a condom demonstration)	82.0 (591)	3.37 [1.93-5.88]	<0.001	2.45 [1.37-4.39]	0.003	1.00 [0.40-2.48]	0.992

Duration since first contacted by intervention staff Has not been contacted Less than one year (greater than zero) One year Two to three years **Test for trends**	15.4 (143) 36.1 (240) 28.0 (198) 20.6 (154)	1.0 (ref) 3.38 [1.65-6.92] 2.08 [1.04-4.18] 3.47 [1.64-7.33]	0.004 **0.058**	1.0 (ref) 1.69 [0.94-3.04] 2.37 [1.23 -4.55] 2.85 [1.45-5.59]	0.012 **0.004**	1.0 (ref) 0.65 [0.19-2.29] 1.41 [0.61-3.28] 1.51 [0.65-3.50]	0.464 **0.165**

Number of times contacted by intervention staff Zero Five or fewer (greater than zero) Greater than five **Test for trends**	15.1 (146) 63.2 (486) 21.7 (137)	1.0 (ref) 2.55 [1.36-4.78] 3.38 [1.32-8.66]	0.006 **0.075**	1.0 (ref) 2.54 [1.47-4.42] 1.80 [0.62-5.27]	0.003 **0.821**	1.0 (ref) 1.20 [0.53-2.75] 0.85 [0.23-3.15]	0.507 **0.603**

Number of condom demos seen past month by staff Zero One Two Three or greater **Test for trends**	18.0 (160) 23.0 (183) 22.2 (180) 36.8 (228)	1.0 (ref) 1.99 [1.10-3.61] 4.72 [2.28-9.77] 4.48 [1.96-10.28]	<0.001 **0.001**	1.0 (ref) 1.77 [0.90-3.49] 4.51 [2.37-8.60] 2.23 [1.11-4.49]	<0.001 **0.099**	1.0 (ref) 1.09 [0.49-2.43] 2.08 [0.94-4.61] 0.73 [0.21-2.46]	0.130 **0.499**

Condoms used in all occasions of sexual intercourse with clients in the most recent day worked	81.7 (585)						

Consistent condom use with all occasional clients	69.5 (530)						

Consistent condom use with most recent repeat client	57.5 (269)						

Consistent condom use with most recent non-paying partner	31.1 (68)						

Consistent condom use with husband/cohabiting partner	9.6 (40)						

^1^Not all of the outcomes have the same denominator, as the sample was subset to women with different types of partners for each outcome; condoms used in all occasions of sexual intercourse with clients in the most recent day worked has a smaller denominator than consistent condom use with all occasional clients because of missing data in the former outcome.

^2^Consistent condom use is defined as reporting always (100%) using condoms.

^3^The total N for each factor may not add up to 775 due to missing values.

### Relationship between the intervention and condom use

#### Commercial sex clients

**Figure 1:**
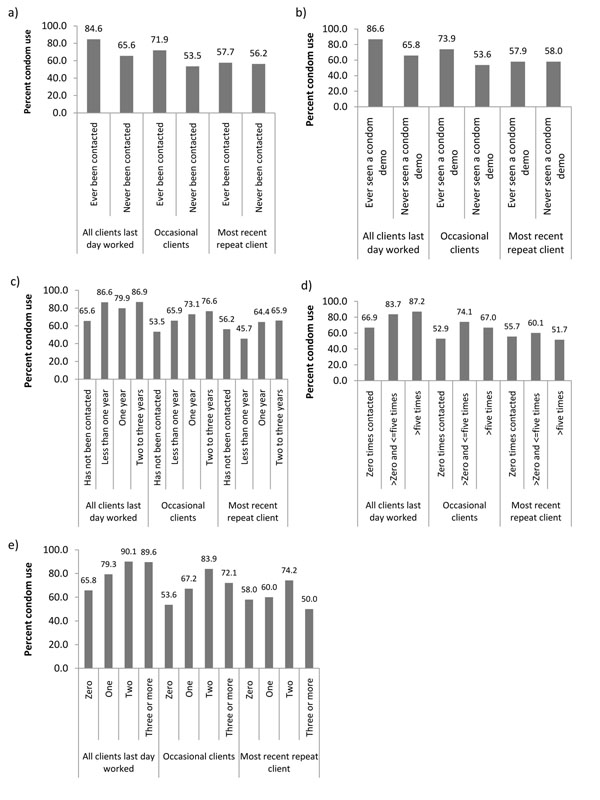
Relationship between indicators of intervention exposure and consistent condom use (CCU). These include CCU with all commercial clients of female sex workers (FSWs) in the most recent day worked, CCU with occasional clients and CCU with the most recent repeat client, based on the results of special behavioural surveys in Karnataka state: (a) CCU vs. ever been contacted by intervention staff; (b) CCU vs. ever seen a condom demonstration by intervention staff; (c) CCU vs. time since first contacted by programme staff; (d) CCU vs. number of times contacted by staff in the past month; and (e) CCU vs. number of condom demonstrations by staff observed by FSWs in the past month.

The sample of 775 FSWs all reported having occasional clients. Of these women, 433 had repeat clients. Overall, 585 (81.7%) of FSWs reported CCU with all clients in the most recent day worked, 530 (69.5%) women reported CCU with current occasional clients and 269 (57.5%) women reported CCU with their most recent repeat client. CCU with all clients was higher among FSWs who had ever been contacted by intervention staff compared to those who had not (84.6% versus 65.6%), as was CCU with occasional clients (71.9% versus 53.5%) (Figure 1a). The same patterns were observed for FSWs who had ever been given condoms by intervention staff compared to those who had not (CCU with all clients: 86.6% versus 65.8%; CCU with occasional clients: 73.9% versus 53.6%) (Figure 1b). CCU was approximately the same with the most recent repeat client for women who had ever been contacted by intervention staff compared to those who had not (57.7% versus 56.2%) and for those who had ever seen a condom demonstration (57.9% versus 58.0%) compared to those who had not.

CCU with all clients in the most recent day worked, with occasional clients and with the most recent repeat client, increased overall as the duration of time since first contact by intervention staff increased, but only steadily increased with increased duration for CCU with occasional clients (Figure 1c). CCU with all clients increased steadily as the number of times contacted by staff in the past month increased (Figure 1d). CCU was highest with occasional clients and with the most recent repeat client among women who had been contacted <5 times (relative to women who had never been contacted or who had been contacted 5+ times). Finally, and consistent with the previous outcome, CCU with all clients in the last day worked, with occasional clients and the most recent repeat client increased with the number of condom demonstrations observed in the last month, but levelled off and decreased (substantially for the two latter outcomes) at two condom demonstrations in the last month (Figure 1e).

In bivariate analysis, all five intervention variables were significantly associated (on a P<0.10 significance level) with CCU with all clients in the most recent day worked and CCU with occasional clients (Table 1). Other explanatory variables that were significant on a P<0.10-level, or included in multivariable models a priori, are also listed in Table 1. In multivariable analysis (Table 2), after adjusting for social and environmental factors, the odds of CCU with all clients in the most recent day worked and CCU with occasional clients were 6.3-fold [95% CIs 2.8-14.5] and 2.3-fold [95% CIs: 1.3-4.1] higher among FSWs who had ever been contacted by intervention staff and 4.9-fold [95% CIs 2.6-9.3] and 2.3-fold [95% CIs: 1.3-4.1] higher among FSWs who had ever been given condoms by intervention staff, compared to those who never had been. None of the intervention exposure variables were significantly associated with CCU with the most recent repeat client in bivariate or multivariable analysis (Tables 1 and 2).

**Table 2:** Multivariable associations^1^ (adjusted odds ratios [AOR]) and 95% confidence intervals (95% CIs): multivariable associations between social, environmental and intervention exposure factors and consistent condom use with commercial sex clients. Five models (MODEL1-MODEL5) were constructed for each of the five explanatory variables for intervention exposure and each outcome, for 15 models total.

MODEL^1^		OUTCOME: Consistent condom^2^ use within different sexual partnerships					
		
		Condoms used in each occasion of sexual intercourse with clients in the most recent day worked		Consistent condom use with occasional clients		Consistent condom use with most recent repeat client	
	
	INTERVENTION EXPOSURE	AOR [95% CIs]	P	AOR [95% CIs]	P	AOR [95% CIs]	P
1	Ever contacted by intervention staff (versus not ever contacted)	6.32 [2.76-14.47]	<0.001	2.30 [1.30-4.08]	0.006	1.07 [0.34-3.33]	0.993

2	Had a condom demonstration by intervention staff (versus never had a condom demonstration)	4.89 [2.57-9.30]	<0.001	2.30 [1.30-4.07]	0.009	0.88 [0.29-2.66]	0.812

3	Duration since first contacted by intervention staff Has not been contacted Less than one year (greater than zero) One year Two-three years **Test for trends**	1.0 (ref) 5.80 [2.54-13.29] 5.38 [2.06-14.06] 11.76 [3.80 -36.33]	<0.001 <0.001 <0.001 **0.001**	1.0 (ref) 1.77 [0.98-3.20] 2.72 [1.33-5.56] 3.71 [1.55-8.87]	0.095 0.011 0.005 **0.006**	1.0 (ref) 0.65 [0.19-2.19] 1.53 [0.51-4.65] 2.55 [0.79-8.23]	0.487 0.449 0.118 /

4	Number of times contacted by intervention staff Zero Five or fewer (greater than zero) Greater than five **Test for trends**	1.0 (ref) 5.10 [2.28 -11.43] 7.66 [2.27-25.87]	<0.001 0.010 **0.047**	1.0 (ref) 2.65 [1.42-4.96] 1.99 [0.78-5.08]	0.002 0.153 /	1.0 (ref) 1.12 [0.36-3.51] 0.87 [0.21-3.60]	0.847 0.844 /

5	Number of condom demos by staff seen past month Zero One Two Three or greater **Test for trends**	1.0 (ref) 3.41 [1.69-6.86] 7.76 [3.08-19.54] 4.88 [2.17-10.97]	<0.001 <0.001 <0.001 **0.004**	1.0 (ref) 1.48 [0.72-3.04] 4.59 [2.26-9.29] 2.13 [1.14-3.97]	0.283 <0.001 0.018 **0.026**	1.0 (ref) 1.08 [0.42-2.76] 1.97 [0.80-4.87] 0.70 [0.21-2.35]	0.875 0.143 0.561 /

^1^ Models were all adjusted for variables that were included a priori and variables that were significantly associated with each outcome on the p<0.10-level in bivariate analysis. For all three outcomes of condom use, a priori variables included typology of sex work (place of solicitation) and district; for condoms used in each occasion of sexual intercourse with clients in the most recent day worked, models were also adjusted by marital status, age and age at first sex; for condom use with occasional clients, models were also adjusted by age, marital status, literacy, age at first sex work; for condom use with most recent repeat client, models were also adjusted by education.

^2^ Consistent condom use is defined as reporting always (100%) using condoms.

/ Test for trend not significant in bivariate analysis, and was not tested in multivariable models.

In bivariate analysis testing for trends, CCU with all clients in the most recent day worked was significantly associated with an increased duration since first contacted by intervention staff, the number of times contacted by intervention staff, and the number of condom demonstrations seen by staff in the last month. CCU with occasional clients was significantly associated with an increased duration since first being contacted by intervention staff, and the number of condom demonstrations seen by staff in the last month. In multivariable analysis, significant tests for trends indicated a dose-response relationship between CCU with all clients in the most recent day worked and CCU with occasional clients, and increased duration since first being contacted by staff (*P*=0.001, *P*=0.006 respectively). For both of these outcomes, significant tests for trends also indicated a dose-response relationship between CCU with all clients in the most recent day worked and CCU with occasional clients and increased numbers of condom demonstrations witnessed (*P*=0.004 and *P*=0.026 respectively). Finally, a dose-response relationship was also observed between CCU with all clients and number of times contacted by staff (*P*=0.047).

#### Non-commercial partners

**Figure 2:**
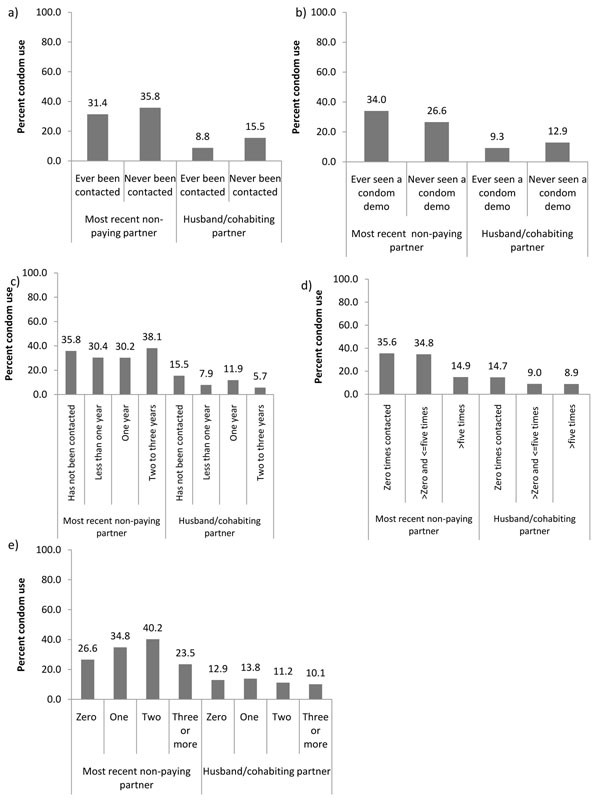
Relationship between indicators of intervention exposure and consistent condom use (CCU). These included CCU with the most recent non-paying partner of female sex workers (FSWs) (who was neither the husband nor the main cohabiting partner) and FSWs’ husband or cohabiting partner, based on the results of special behavioural surveys in Karnataka state: (a) CCU vs. ever been contacted by intervention staff; (b) CCU vs. ever seen a condom demonstration by intervention staff; (c) CCU vs. time since first contacted by programme staff; (d) CCU vs. number of times contacted by staff in the past month; (e) CCU vs. number of condom demonstrations by staff observed by FSWs in the past month.

Of the total sample, 226 FSWs reported having a non-paying sexual partner in the last year (who was neither the husband nor main cohabiting partner) and 354 had a husband or cohabiting partner. Overall, 68 (31.1%) and 40 (9.6%) reported CCU with their most recent non-paying partner and their husband or cohabiting partner respectively (Table 1). In contrast to CCU with all clients and with occasional clients, CCU with their most recent non-paying partner was higher among FSWs who had never been contacted by intervention staff compared to those who had been contacted (35.8% versus 31.4%); the same was true for CCU with their husband or cohabiting partner (15.5% versus 8.8%). CCU with their most recent non-paying partner was higher for those who had seen a condom demonstration compared with those who had not (34.0% versus 26.6%), while CCU with their husband or cohabiting partner was higher for those who had never seen a condom demonstration compared to those who had (12.9% versus 9.3%) (Figures 2a-2b). Figure 2c demonstrates how CCU with both types of partners decreased and then increased as the duration of time since first contacted by intervention staff increased. CCU with both their most recent non-paying partner and CCU with their husband or cohabiting partner decreased as the number of times contacted by staff in the past month increased (Figure 2d). CCU with the most recent non-paying partner initially increased as the number of condom demonstrations witnessed increased, and then dropped by almost half for women who had seen three or more demonstrations in the past month. CCU with the husband or cohabiting partner increase slightly for one compared to zero demonstrations, then decreased steadily with increasing number of condom demonstrations (Figure 2e).

In bivariate logistic regression, only the variable number of times contacted by intervention staff was significantly associated with CCU with FSWs’ most recent non-paying partner (on a P<0.10 significance level). In multivariable analysis, after adjusting for social and environmental factors, none of the intervention exposure variables were significantly associated with CCU with their non-paying partner or husband or cohabiting partner (Table 3). In bivariate analysis testing for trends, CCU with FSWs’ most recent non-paying partner was inversely associated with the number of times contacted by intervention staff and CCU with their husband or cohabiting partner was inversely associated with the number of condom demonstrations by intervention staff. In multivariable analysis testing for trends, CCU with FSWs’ husband or cohabiting partner remained inversely significantly associated with the number of condom demonstrations by intervention staff (P=0.05).

**Table 3:** Multivariable associations^1^ (adjusted odds ratios [AOR]) and 95% confidence intervals (95% CIs): multivariate associations between social, environmental and intervention exposure factors and condom use with non-commercial partners. Five models (MODEL1-MODEL5) were constructed for each of the five explanatory variables for intervention exposure and each of the two outcomes, for 10 models total.

		Consistent condom use^2^ with most recent non-paying partner		Consistent condom use^2^ with husband or cohabiting partner	
		
		AOR [95% CIs]	P	AOR [95% CIs]	P
1	Ever contacted by intervention staff (versus not ever contacted)	1.40 [0.47-4.18]	0.542	0.35 [0.11-1.16]	0.085

2	Had a condom demonstration by intervention staff (versus never had a condom demonstration)	1.72 [0.60-4.91]	0.311	0.50 [0.17-1.43]	0.194

3	Duration since first contacted by intervention staff Has not been contacted Less than one year (greater than zero) One year Two-three years **Test for trends**	1.0 (ref) 1.05 [0.34-3.29] 1.52 [0.43-5.40] 2.74 [0.64-11.74]	0.930 0.515 0.173 /	1.0 (ref) 0.39 [0.11-1.44] 0.48 [0.13-1.78] 0.20 [0.03-1.43]	0.156 0.272 0.108 /

4	Number of times contacted by intervention staff Zero Five or fewer (greater than zero) Greater than five **Test for trends**	1.0 (ref) 1.66 [0.56-4.93] 0.43 [0.09-2.09]	0.358 0.293 **0.146**	1.0 (ref) 0.42 [0.13-1.36] 0.43 [0.11-1.66]	0.146 0.219 /

5	Number of condom demos by staff seen past month Zero One Two Three or greater **Test for trends**	1.0 (ref) 3.43 [0.95-12.48] 3.39 [0.89-12.97] 1.02 [0.28-3.67]	0.061 0.074 0.979 /	1.0 (ref) 0.86 [0.23-3.15] 0.64 [0.18-2.27] 0.33 [0.10-1.00]	0.815 0.491 0.072 **0.045**

^1^ Models were all adjusted for variables that were included a priori and variables that were significantly associated with each outcome on the p<0.10-level in bivariate analysis. For all three outcomes, a priori variables included district and typology of sex work (place of solicitation); for condom use with the husband or cohabiting partners, models were also adjusted by age at first sex work.

^2^ Consistent condom use is defined as reporting always (100%) using condoms.

/ Test for trend not significant in bivariate analysis, and was not tested in multivariable models.

## Discussion

The results from our analysis suggest that exposure to a large-scale HIV prevention initiative in Karnataka, India, was associated with higher reported consistent condom use (CCU) among women engaged in sex work with their commercial sex clients. After adjusting for social and environmental factors, a strong independent association was observed between CCU with all clients in the most recent day worked and CCU with occasional clients, and five measures of intervention exposure. Moreover, a significant dose-response relationship was observed between these two outcomes and increased duration since first contacted by intervention staff, as well as number of condom demonstrations seen by staff in the last month. There was also a significant dose-response relationship observed between CCU with all clients and the number of times contacted by staff in the past month. In multivariable analysis, intervention exposure was not significantly associated with increased CCU with FSWs’ most recent repeat commercial client, their most recent non-paying partner or their husband or cohabiting partner.

The association between increased intervention exposure and increased CCU with all clients likely reflects higher condom use with occasional clients, which constitute the majority of commercial clients in Karnataka. On a micro-level, condom use with occasional clients likely improved due to regular contact between FSWs and peer outreach workers (i.e., members of local sex worker communities), who were responsible for providing condoms to FSWs, giving demonstrations of correct condom use and facilitating conversations about risk and vulnerability [15]. Of note, our exposure variables measuring contacts by peers were not independent of our variables measuring condom demonstrations by peers. Although intervention exposure variables could not directly capture the influence of community involvement or mobilization strategies, peers also encouraged membership in community groups and were proponents of community mobilization, which is intended to facilitate condom negotiation by FSWs and use with clients through both individual-level and collective empowerment and agency [14,18]. Interestingly, CCU with clients in this analysis was highest for FSWs who had seen two condom demonstrations by staff in the previous month and lower for FSWs who had seen three or more. CCU with occasional clients and repeat clients was also higher among FSWs who had been contacted <5 times compared to those who had been contacted 5+ times. These results could suggest that there may be a point where increased contact by staff or education about correct condom use by intervention staff will not improve condom use [29]. Resources may be better directed to other features of the intervention if additional increases in condom use are to be observed. CCU was found to steadily increase with increased duration since first contacted by the intervention. This effect may not have levelled off (as with the previous two intervention exposure variables) over time due to the limited amount of time since the intervention began in some districts (varied from 1.5-2.5 years). Condom use may also have naturally increased over time in southern India (reflected in the duration since first contacted by staff) albeit likely at slower rates than in if the intervention was not present. Condom use may not have reached 100% in all commercial sex acts due to the timing of survey data collection (e.g., condom use may still increase with increased exposure to the intervention). There may also be groups of highly vulnerable FSWs who may be unable to negotiate condom use with all clients who refuse to wear condoms, even if exposed to the intervention. Condom use with commercial clients was relatively high for those FSWs who reported that they were not exposed to the intervention. This may be due to the presence of other HIV prevention programmes in place prior to Avahan. SBS surveys were also implemented 7-19 months after Avahan was introduced in different districts, and an independent analysis retrospectively assessing condom use confirmed that condom use increased notably after Avahan was introduced [30]. Condom use may also be high due to the indirect impacts of Avahan (e.g., through increased peer awareness of condoms or increased condom availability [31]). Improved condom availability was also a key feature of the intervention [31]. This facilitated condom use simply by increasing access, but also likely by increasing social acceptance of condoms through increased visibility and presence. Other interventions incorporating these program elements have shown success in improving condom use among FSWs [10,11,32].

Results from this study are supported by other studies showing similar results. These include observational studies suggesting that condom use as reported by clients [33] and FSWs [30,34] increased after the introduction of the intervention, as well as studies suggesting that condom availability to FSWs increased substantially since the intervention began [31]. Increases in condom use among high-risk groups could have important implications for HIV and STI prevalence in Karnataka. Sentinel surveillance and observational studies have found decreasing trends in terms of HIV and STIs among FSWs in Karnataka state since the intervention was introduced [34,35]. Moreover, results are also consistent with mathematical modelling indicating that the increase in condom use after initiation of the intervention was consistent with decreasing HIV epidemiological trends over multiple rounds of survey data collection in Karnataka state [36,37]. There is evidence to suggest that increased condom levels can be sustained over time in this population [34]. Nevertheless, continued monitoring of condom use levels and assessments of the impact of observed increases in condom use on reducing HIV and STIs is important for a long-term and comprehensive understanding of the impact of the intervention.

While a higher probability of CCU with all clients in the most recent day worked and occasional clients was observed for FSWs with increased exposure to the intervention, the same patterns were not observed among FSWs for CCU with their most recent repeat client, non-paying partner and their husband or cohabiting partner. Moreover, CCU with FSWs’ husband or cohabiting partner decreased significantly with increasing numbers of condom demonstrations seen by intervention staff in the previous month, when testing for trends. It is not clear why this was observed, but of note, CCU with FSWs’ husband or cohabiting partner was very low and the absolute proportions did not vary substantially according to the number of condom demonstrations seen (10.1% to 13.8%). The reasons for low condom use within non-commercial and regular commercial sexual partnerships of FSWs are complex. These may include power disparities that favour the male partner [4,38,39], including an economic dependence on longer-term male partners [40,41]. The use of condoms may not be acceptable in non-commercial relationships, if there is greater longevity, trust and intimacy within the partnership; the use of condoms may also be perceived as a symbol of infidelity and foster mistrust [42]. Women may agree to not use condoms with repeat clients in exchange for these partners providing a stable form of longer-term income or because they feel they can assess if their partner is not infected with HIV after they have seen him several times. Further research is required to better understand condom use with non-commercial and regular commercial partners of FSWs; in particular, understanding gender-based interpersonal factors that influence condom use and preferences by both partners, as well as environmental factors (e.g. favourable societal views of condoms) that could be incorporated into interventions to increase condom use. The female condom could play an increasing role in HIV prevention within these partnerships. In addition, recent promising findings of the effectiveness of microbicides indicate that microbicides could play an important role in HIV prevention as an alternative to condoms for women whose partner does not use condoms [43].

### 

There are several limitations to this study. This study is based on self-reported data from cross-sectional surveys collected in three districts and is not experimental in design. This study was based on data collected only from FSWs and should be considered alongside studies that show consistent results in other populations (e.g., clients), using other data sources to assess exposure to the intervention, and in similar settings. The condom use outcomes used in this study did not specify a timeframe. However, the questions were intended to capture recent (e.g., condom use with the last 10 clients) or current average behaviour. We relied on self-reported answers to questions that may be perceived as sensitive (e.g. consistency of condom use), and the questions are therefore susceptible to social desirability bias [44]. This may have overestimated the relationship between intervention exposure and CCU, and it is possible that women with increased condom use are more likely or able to be accessed by intervention-related services and programs rather than the other way around. However, we believe that this is unlikely, since the total sample size was relatively large, particularly for a marginalized and hidden population, and the cluster sampling design was used to make the sample as representative as possible, with complex survey methods accounting for the sampling design. Additionally, our results suggest not only a relationship between exposure to the intervention and increased condom use, but a dose-response relationship between increased exposure to the intervention and increased condom use with all clients and occasional clients.

### 

The impact of increases in condom use among FSWs and their clients on the HIV epidemic in southern India should continue to be assessed. Large-scale HIV prevention programs targeted at groups with high HIV risk could in theory also have an indirect impact on HIV prevalence within general populations [45]; however, results from mathematical modelling suggest that the current observed decrease in antenatal clinic HIV prevalence in India was likely not caused by the FSW-targeted intervention, although it is likely too early to assess the impact of the intervention on bridge groups or groups with low risk for HIV [46]. Since many clients of FSWs have other longer-term and/or non-paying partners such as wives, as well as other FSW partners, and since condom use is low in these partnerships, clients provide an important transmission “bridge” between FSWs and the general population [45,47]. Since condom use tends to be relatively low among FSWs’ repeat clients and non-paying partnerships, these partners can also provide a transmission bridge *to* FSWs [48].

If the intervention’s influence on condom use varies by type of commercial sex client (e.g. occasional compared to repeat clients) *and* the patterns of sexual structure vary geographically (e.g., districts such as Bangalore have higher fractions of occasional clients and lower numbers of repeat clients per month), we would expect to observe different intervention effects across the three districts in Karnataka. CCU with non-paying partners was also much lower in Bangalore (12.8%) compared to the other two districts (45.7% and 41.6% respectively), indicating that the importance of these partnerships in this district may be more pronounced, and should be considered in this district more than others when planning interventions. Exploring the relative role of different patterns of FSW-client sexual structure and variation in the numbers of different types of partners on HIV transmission in Karnataka, India, using simulation studies, would be useful to further improve the impact of the intervention [3,24,49-51].

## Conclusions

In summary, study findings suggest that the exposure to a large-scale HIV preventive intervention among FSWs was associated with increased condom use with occasional clients, with a dose-response relationship, but that it did not seem to influence condom use with repeat clients, non-paying partners and with the husband or cohabiting partner. Future research should be directed toward understanding why condom use remains relatively low with non-commercial partners and new strategies should be investigated and developed specifically to increase condom use by these partners.

## Competing interests

The authors declare that they have no competing interests.

## Authors' contributions

KND contributed to the conceptual design of the study, conducted the study and the analysis and drafted the manuscript; MCB, CML, PV, MP and MA participated in the conceptual design of the study and coordination, made substantial contributions in the interpretation of data; JS, MWT, SM and JB made substantial contributions in the interpretation of data and critically revised the manuscript for important intellectual content; BMR, KG, SR, RW made substantial contributions to the acquisition and management of the data. All authors read and approved the final manuscript.
